# Public Emotions and Rumors Spread During the COVID-19 Epidemic in China: Web-Based Correlation Study

**DOI:** 10.2196/21933

**Published:** 2020-11-25

**Authors:** Wei Dong, Jinhu Tao, Xiaolin Xia, Lin Ye, Hanli Xu, Peiye Jiang, Yangyang Liu

**Affiliations:** 1 School of Education Tianjin University Tianjin China; 2 School of Media and Communication Shanghai Jiaotong University Shanghai China; 3 College of Life Sciences and Bioengineering, School of Science Beijing Jiaotong University Beijing China; 4 Office of International Cooperation and Exchanges Nanjing University Nanjing China

**Keywords:** public emotions, rumor, infodemic, infodemiology, infoveillance, China, COVID-19

## Abstract

**Background:**

Various online rumors have led to inappropriate behaviors among the public in response to the COVID-19 epidemic in China. These rumors adversely affect people’s physical and mental health. Therefore, a better understanding of the relationship between public emotions and rumors during the epidemic may help generate useful strategies for guiding public emotions and dispelling rumors.

**Objective:**

This study aimed to explore whether public emotions are related to the dissemination of online rumors in the context of COVID-19.

**Methods:**

We used the web-crawling tool Scrapy to gather data published by People’s Daily on Sina Weibo, a popular social media platform in China, after January 8, 2020. Netizens’ comments under each Weibo post were collected. Nearly 1 million comments thus collected were divided into 5 categories: happiness, sadness, anger, fear, and neutral, based on the underlying emotional information identified and extracted from the comments by using a manual identification process. Data on rumors spread online were collected through Tencent’s Jiaozhen platform. Time-lagged cross-correlation analyses were performed to examine the relationship between public emotions and rumors.

**Results:**

Our results indicated that the angrier the public felt, the more rumors there would likely be (r=0.48, *P*<.001). Similar results were observed for the relationship between fear and rumors (r=0.51, *P*<.001) and between sadness and rumors (r=0.47, *P*<.001). Furthermore, we found a positive correlation between happiness and rumors, with happiness lagging the emergence of rumors by 1 day (r=0.56, *P*<.001). In addition, our data showed a significant positive correlation between fear and fearful rumors (r=0.34, *P*=.02).

**Conclusions:**

Our findings confirm that public emotions are related to the rumors spread online in the context of COVID-19 in China. Moreover, these findings provide several suggestions, such as the use of web-based monitoring methods, for relevant authorities and policy makers to guide public emotions and behavior during this public health emergency.

## Introduction

In December 2019, the earliest cases of COVID-19 were reported in Wuhan, Hubei Province, China. On January 23, 2020, several cities in Hubei Province were quarantined in an attempt to slow down community transmission of the disease. On January 30, 2020, the World Health Organization officially announced that the COVID-19 outbreak was a public health emergency of international concern [1]. As COVID-19 has become a serious global problem, people all over the world are faced with the risk of infection; this has caused a change in people’s behaviors as well as fluctuations in their emotions [2]. During COVID-19 outbreaks in different parts of the world, there was a proliferation of emotional comments and occasional rumors posted on the internet. Although a number of epidemiological and clinical studies have been performed thus far, few studies have examined public emotional response, and to our knowledge, no study has investigated whether public emotions are related to the occasional online rumors spread in the context of COVID-19. In this study, we seek to contribute to the literature by addressing this gap in the existing research.

Rumors have abounded since ancient times, but they have been the focus of researchers only since the Second World War [3]. In a pioneer study, Prasad [4] proposed that rumors are spread due to the anxiety and fear of unknown things in a disaster. Allport and Postman [5] suggested that the spread of rumors is a process of releasing emotions by telling stories to others. The lack of barriers of the internet enables individuals to participate in online discussions anonymously, which creates a favorable environment for the spread of rumors [6]. Some empirical studies have suggested that among the messages forwarded on the internet by users, those with emotional elements (anger, sadness, or anxiety) were 36.7% more frequent than those without emotional elements [7] and that people are more likely to believe online rumors with the same type of emotion that they currently feel. That is, angry people are more receptive to anger-related rumors [8]. However, until now, online rumors have not been extensively researched. All prior research has focused on analyzing rumors generated due to anxiety and fear. It is unclear whether other emotions are related to the spread of rumors. Additionally, no empirical studies have examined whether there is a significant correlation between emotions and rumors in the context of major public health emergencies.

In the context of COVID-19, various online rumors have led to inappropriate behaviors among people in response to the epidemic, which have adversely affected people’s physical and mental health [9]. We believe that the findings of this study can provide some useful strategies to guide public emotions and dismiss rumors, in an effort to fight this global crisis.

Previous studies on public emotions during major public health emergencies have found that people usually experience negative emotions such as panic, anxiety, anger, grief, and disgust [10,11], likely due to the illnesses and deaths that occur during these emergencies. For example, 17 years ago, a study on SARS found that tension, helplessness, panic, and anger were significantly related to an increase in the number of SARS cases [12]. Some other studies also showed that public anxiety changed with the development of the epidemic (eg, avian influenza A [H7N9] or influenza A [H1N1]) [13,14]. In line with some previous studies that used data from different sources [15,16], we aimed to examine the dynamic relationship between the public’s emotions and rumors in the context of COVID-19, by using data from China’s mainstream internet media platforms (Sina Weibo and Tencent). According to a report released by the China Internet Network Information Center, as of March 2020, there were 904 million internet users and the internet penetration rate in China had reached 64.5% [17]. Hence, the data obtained through the online responses from netizens are considered to be representative of the public’s response to major health emergencies [18]. Moreover, social media surveillance can provide real-time information on the public’s response; solve the problem of underrepresentation of samples; and avoid recall or reporting bias caused by personal observation, collection, and recording [19,20]. According to cognitive dissonance theory [21], rumors may serve as a channel for the public to reduce or eliminate cognitive dissonance caused by emotions. In this study, we aimed to explore whether the public’s emotions are related to the generation and dissemination of rumors in the context of COVID-19.

## Methods

### Epidemiological Data

On January 20, 2020, China launched a monitoring and quarantine system to record daily information about COVID-19 cases. The epidemiological data, issued by the National Health Commission of People’s Republic of China (NHC), were collected at the grassroots level on a daily basis. For this study, we used the epidemiological data (specifically, the daily number of newly confirmed COVID-19 cases) from all provincial-level regions of China that were released by the NHC from January 20 to March 10, 2020.

### Data on Public Emotions

Sina Weibo, the Chinese version of Twitter, is the largest social media platform in China. People’s Daily, with more than 116 million followers on Weibo, is one of the most influential and authoritative news media on Sina Weibo. In this study, we used the web crawler Scrapy to gather relevant data published by People’s Daily on Weibo between January 20 and March 8, 2020, as well as netizens’ comments under each Weibo post. Millions of real-time comment texts contain substantial emotional information; we identified and extracted these public emotions from the comments collected.

Although many sentiment analysis tools based on natural language processing or machine learning [22,23] are able to automatically extract emotions from the text by utilizing less time and labor, the accuracy of machine identification is lower than that of manual identification. Some simple classifications, such as positive or negative, can be detected through machine identification; however, for more subtle discrete emotions, such as anger, fear, and happiness, manual identification would yield better accuracy. Thus, we adopted manual identification methods in this study. Recently, Jack et al [24] reported that people universally experience only 4 basic emotions: happiness, sadness, fear, and anger. In this study, public emotions were divided into 5 categories: the 4 abovementioned basic human emotions (ie, happiness, sadness, fear, and anger) and “neutral” (ie, representing no emotions). A total of 143 trained research assistants participated in the manual identification process. Each online comment was identified and classified by at least 3 trained research assistants, and conflicting classification results were resolved by a majority consensus to arrive at the final classification. The interrater reliability of the coders was “substantial” (κ=0.77) [25].

### Data on Emotional Rumors

Tencent’s Jiaozhen [26] is the first network-wide, professional, and timely fact-checking platform in China that checks internet news that has been widely circulated and dispels rumors on a daily basis. Data on online rumors about COVID-19 disseminated between January 20 and March 10, 2020, were collected through this platform, and manual identification was performed to identify all kinds of emotional rumors spread during this period.

### Data Calibration and Statistical Analysis

**Figure 1:**
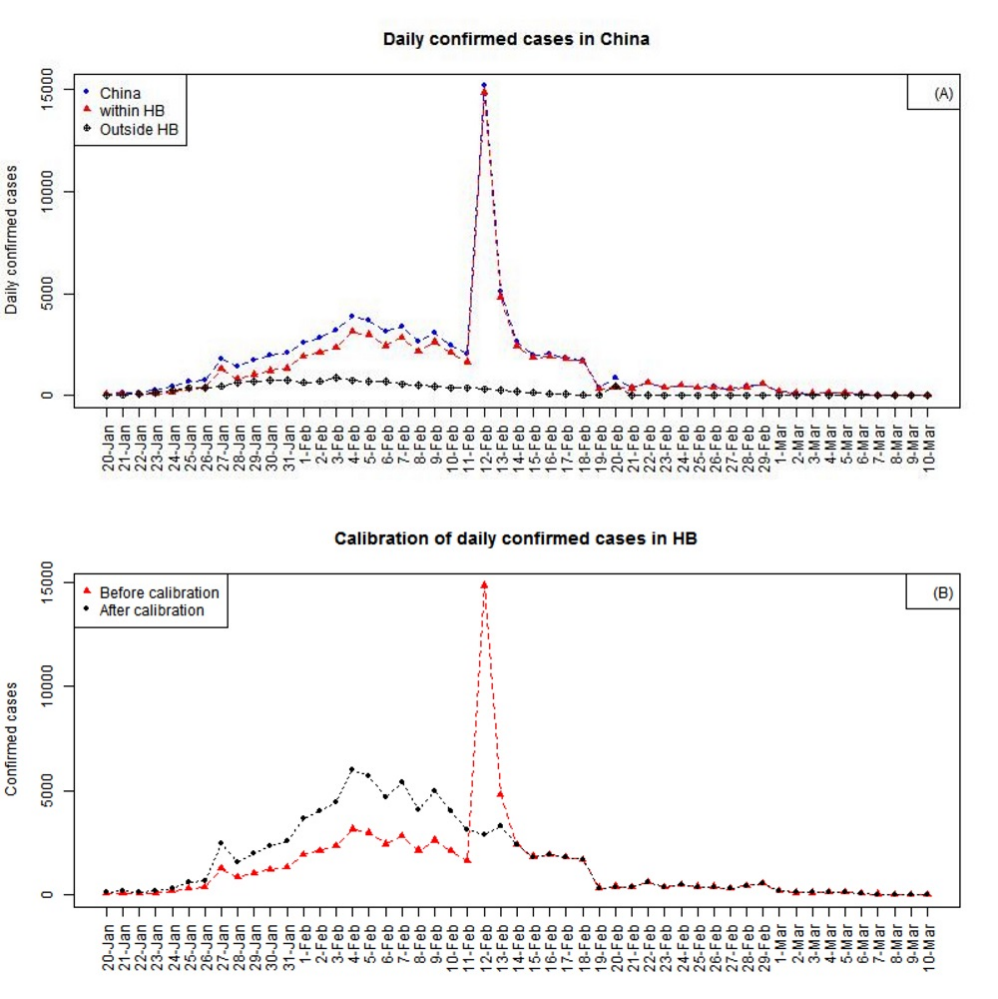
(A) Daily confirmed cases of COVID-19 in China. Blue dots represent numbers for the whole country, red triangles represent numbers for Hubei Province, and black circles represent numbers for other provinces. (B) Calibration of the daily number of confirmed cases in Hubei Province. Red triangles represent the daily number of confirmed cases in the province before calibration, and black dots represent the daily number of confirmed cases in the province after calibration. The sharp peak disappeared after calibration. HB: Hubei Province.

Figure 1A shows that the daily number of newly confirmed cases in Hubei Province suddenly surged on February 12, 2020, when the diagnosis criteria were revised. Thereafter, the COVID-19 diagnosis criteria used in Hubei was consistent with those outside the province. Therefore, the epidemiological data of newly confirmed cases in Hubei that were collected before February 14 were calibrated before the follow-up analysis.

According to the Hubei Health Committee, on February 12 and 13, 2020, the ratios of COVID-19 cases that were clinically diagnosed and those that were detected based on nucleic acid testing were 8.44 (13,332/1580) and 1.79 (3095/1728), respectively. Most of these cases were a result of long-term accumulation of suspected cases. The decline in the number of clinically diagnosed cases on February 13, 2020, suggested that the cumulative suspected cases had been processed within those 2 days. The corresponding ratios of COVID-19 cases that were clinically diagnosed and those detected via nucleic acid testing on February 14 and 15, 2020, were 0.89 (1138/1282) and 0.93 (888/955), which average at 0.91. Therefore, from January 20 to February 13, 2020, the calibrated daily number of newly confirmed cases (as shown in Figure 1B) was the number of cases confirmed by nucleic acid detection multiplied by (1+0.91).

As the data on daily epidemic situations and emotional comments published on Weibo showed an exponential distribution, the data were log-transformed to stabilize the variance of time-series before conducting further analysis.

We used time-lagged cross-correlation to examine the relationship between public emotions and the total number of rumors and that between public emotions and the rumors with different types of emotions. We also used Pearson correlation coefficient to examine these relationships, with the maximum time lag of 10 days and a *P* value smaller than .05 as a threshold.

## Results

### Rumors in the Context of the COVID-19 Epidemic

**Figure 2:**
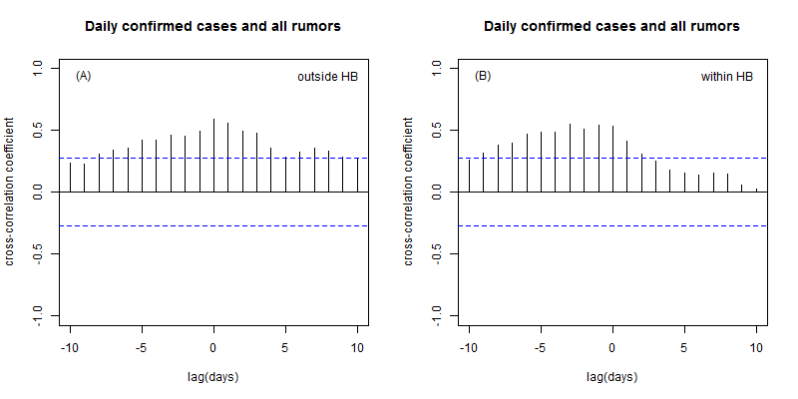
(A) Cross-correlation between the daily number of newly confirmed cases outside Hubei Province and all rumors. (B) Cross-correlation between the daily number of newly confirmed cases in Hubei Province and all rumors. Blue dashed lines denote the 95% confidence intervals of the uncorrelated time series. HB: Hubei Province.

Among the 276 rumors collected, 176 (63.8%) were neutral rumors, 62 (22.5%) were fear-related rumors, 19 (6.9%) were happiness-related rumors, 12 (4.4%) were anger-related rumors, and 7 (2.5%) were sadness-related rumors. Figure 2A shows the relationship between the COVID-19 epidemic situation outside Hubei Province and online rumors. The peak correlation was 0.59 (*P*<.001) when the lag value was 0 days, indicating that the daily number of newly confirmed cases was significantly positively correlated with rumors, with changes occurring simultaneously. Figure 2B shows the relationship between the COVID-19 epidemic situation in Hubei Province and online rumors. The peak correlation was 0.55 (*P*<.001) with a lag of -1 day, which suggests that the total number of rumors changed 1 day in advance of the change in the number of cases in Hubei Province. In addition, the epidemic situation within and outside Hubei Province was not significantly related to the 4 types of emotional rumors.

### Relationship Between Public Emotions and Rumors

**Figure 3:**
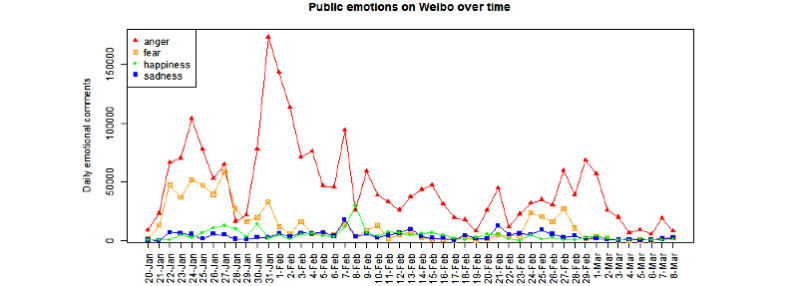
Trend of public emotions expressed on Weibo in the context of COVID-19 during the study period.

During the study period, anger (2,248,362/17,328,675, 12.97%) dominated public emotions on the internet, followed by fear (627,407/17,328,675, 3.62%), happiness (216,072/17,328,675, 1.25%), and sadness (195,708/17,328,675, 1.13%). Figure 3 presents the trend of public emotions on Weibo, which shows that the number of anger-related and fear-related comments were more than the number of happiness-related and sadness-related comments almost every day.

**Figure 4:**
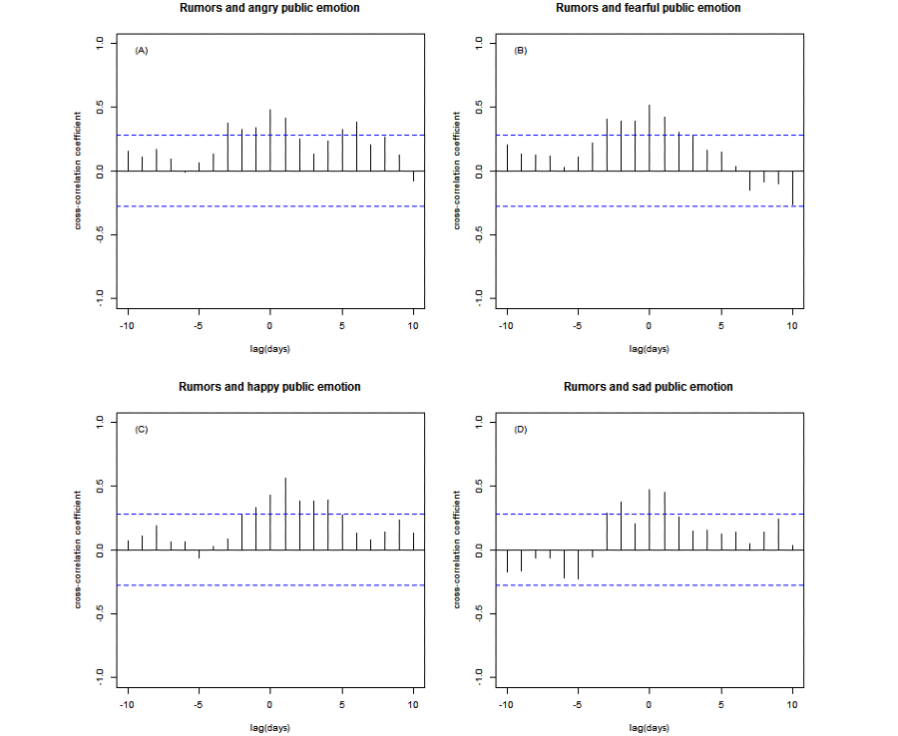
Cross-correlation between emotions and all rumors. Cross-correlation between (A) anger and all rumors, (B) fear and all rumors, (C) happiness and all rumors, and (D) sadness and all rumors. Blue dashed lines denote the 95% confidence intervals of the uncorrelated time series.

Next, we found that the peak correlation of anger and rumors was 0.48 (*P*<.001) with a lag of 0 day (Figure 4A). That is, the angrier the public got, the more rumors there would be. Similarly, the time-lagged analysis between fear and rumors (Figure 4B) and between sadness and rumors (Figure 4D) also indicated significant positive correlations among these 2 emotions and rumors. The results showed the correlations for both these emotions reached their peaks of 0.51 (*P*<.001) and 0.47 (*P*<.001), respectively, with the lag of 0 days. Additionally, a positive correlation was observed between happiness and rumors (r=0.56, *P*<.001, Figure 4C), with a time lag of 1 day. That is, happiness would lag the emergence of rumors by 1 day.

**Figure 5:**
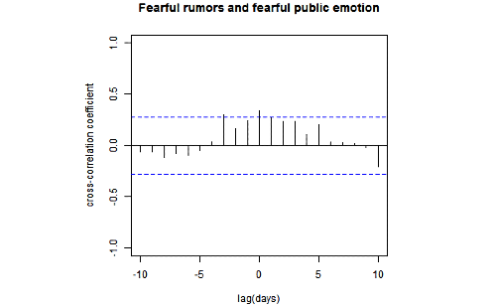
Cross-correlation between fear and fearful rumors. Blue dashed lines denote the 95% confidence intervals of the uncorrelated time series.

Finally, we analyzed the relationship between the 4 types of emotions and different types of rumors. Figure 5 shows that there was a positive correlation only between fear and fearful rumors, with the peak correlation (r=0.34, *P*=.02)) observed at the lag of 0 day.

## Discussion

### Principal Results

To our knowledge, this is the first study to explore the relationship between public emotions and rumors during COVID-19 by using a web-based monitoring method. A large number of comments and rumors related to COVID-19 were obtained from the Weibo account of People’s Daily and Tencent Myth Busters by using the web crawler Scrapy. This web-based monitoring study overcomes the limitations of traditional survey methods and facilitates data-collection in a rapid and real-time manner during outbreak of a public health emergency. In particular, during a large-scale quarantine period, people are more likely to use the internet and social media to acquire and propagate the latest information about the epidemic [7], as well as actively participate in related topic discussions [27]. The internet and social media have become important platforms for real-time monitoring and assessment of changes in public emotions and behaviors. In addition, compared with traditional methods such as survey and telephone interviews, web-based monitoring tends to be more objective and authentic, effectively avoiding measurement errors caused by self-reporting or recalling [14,28]. Manual identification was employed to code emotional categories, which also improved the classification accuracy of public emotion identification [22,23].

In this study, statistically significant relationships were first observed among the daily number of newly confirmed cases and the total number of rumors both within and outside of Hubei Province, which is in line with a previous study that found that the uncertainty of sudden infectious diseases can lead to a large number of rumors [18]. In another study, Li et al [29] analyzed the emotional reactions of 4607 participants through an online survey and found that the COVID-19 did not lead to changes in the frequency of public emotions. However, our study found that the public expressed more anger and fear than happiness and sadness, and there were several large fluctuations in public emotions. Some researchers have suggested that rumors are an outlet for the public to express fear or pressure after a disaster, and spreading rumors could become an effective way for the public to express their emotions such as anger, fear, and sadness [30]. Our findings are in line with these previous findings. The results of our study revealed that three emotions (ie, anger, fear, and sadness) were positively correlated to the total number of rumors, and the emotions and rumors changed synchronously. Moreover, we found that the increase in the happiness emotion tended to lag the emergence of rumors by 1 day. Previous studies have found that public emotions usually transform from negative to positive after the rumors are disproved [31]. In line with these findings, we observed that when the rumors were verified and clarified, there was a proportional increase in the public’s happiness.

Regarding the relationship between the 4 basic human emotions and 4 different types of emotional rumors, our results showed that fearful rumors were positively associated with fear. This result supports the argument presented by of Na et al [8], which claims the consistency between an individual’s emotional state and the emotional information in rumors could increase the prevalence of the rumors. However, our findings regarding anger and anger-related rumors were inconsistent with their conclusion that anger could lead the public to accept anger-related rumors [8]. This difference might be attributed to the differences of sample choice: our study focused on a Chinese sample, whereas Na et al [8] analyzed an American sample; hence, cultural differences in the relationship between public emotions and rumors also need to be further studied.

### Study Limitations

This study, however, has several limitations. First, this study only focused on Sina Weibo for data collection. Other media platforms, such as WeChat in China and Twitter in other countries, were not included to acquire more extensive data. Second, batch-fetching data can only be obtained as a whole; hence, we cannot investigate the change in public emotions at an individual level. In future research, we may use other methods such as surveys or interviews to examine the trajectories of public emotions at an individual level. Third, this study analyzed the relationship between rumors and 4 basic human emotions, and further research could be extended to examine the correlations between rumors and more complex emotions such as anxiety.

### Conclusions

Our findings provide several suggestions for relevant authorities and policy makers in guiding emotions of the public during public health emergencies. First, during a large-scale quarantine period, the authorities can use web-based monitoring methods to identify public emotions and behaviors in real time and provide timely guidance to channel public emotions and behaviors. Second, rumors are a catalyst for public emotions, and disproving them in a timely manner would be helpful to increase positive emotions of the public. Third, our findings showed that fearful rumors were associated with fear. Thus, media platforms should strengthen the monitoring of online rumors, identify and verify emotional rumors in a timely manner, and minimize the spread of fearful rumors to reduce fear among the public.

## Abbreviations

NHC: The National Health Commission of People’s Republic of China
